# Marriage

**Published:** 1858-01

**Authors:** 


### MARRIAGE.

In Massachusetts, during eighteen hundred and fifty-five, among eleven hundred thousand people, there were twelve thousand marriages, four-sevenths of which were among foreigners; and of about thirty-two thousand births, nearly one-half were from foreigners: showing the general fact, that while the foreign-born of Massachusetts are far inferior in numbers to the native-born, they have more marriages, and nearly as many children. This results from the expense of marriage. Too many girls are brought up in total ignorance of domestic duties. Young men see that a wedding involves a maelstrom expense, and the extravagance of hotel or boarding-house life; or of a cook, and housemaid, and semptress, to begin with; then come belongings to match. To meet these calls on his purse, which is only to be replenished by his personal efforts, is only to be done by the ceaseless slavery of work. He "calculates," and declines the undertaking until he can lay up something to begin with. This event, in some instances, is indefinitely postponed; by the time it occurs in others, "mistakes" have been committed, or entangling alliances have diminished the respect for women, and less enduring than hymeneal knots are fabricated, and more easily untied. Let young mothers begin this day to take a wiser lesson; and as they value the social position and future happiness of their daughters, as they would deprecate from them a life of loneliness, an age of neglect, and a tearless burial, let them remember that the education which most befits the sex is that which prepares her to fill with ability, love and dignity, the offices of wife, mother, matron.

The *Newburyport Herald*, with great pertinency, says:

"Our fathers used to tell of the profligacy of Paris; their children tell of the mysteries of New York, a city not far behind any in Europe. And, making proper allowance for size, how far is New York ahead of our other cities and towns? Once was a time, when a wife was "help-meet;" now, in a thousand of cases, you can change the "meet" to "eat," and make it read more truthfully. We boast of our system of education; we have female high schools, female colleges, female medical schools, and female heavens. Our girls are refined, learned, wise; they can sing, dance, play pianos, paint, talk French and Italian, and all the soft languages, write poetry, and love like Venuses. They are ready to be courted at ten years, and can be taken from school and married at fifteen, and divorced at twenty. They make splendid shows on bridal tours, can coquette and flirt at the watering-places, and shine like angels at winter parties. But heaven be kind to the poor wretch who marries in the fashionable circles. What are they at, washing floors? Oh! we forgot; nobody has bare floors now—how vulgar that would be!—What are they at, making bread and boiling beef? Why, how thoughtless we are—to be sure, they will board or have servants. What are they at, mending old clothes? But there we are again; the fashions change so often, that nobody has old clothes but the rag-men and paper-makers now! What are they at, washing babies' faces and pinning up their trowsers? And here is our intolerable stupidity once more; having children is left to the Irish! What lady thinks of having a parcel of children about her now? or if she has them, don't she put them to wet-nurses to begin with, and boarding-schools afterwards?

We have come to a point, where young men hesitate and grow old before they can decide whether they can marry, and afterwards keep clear of bankruptcy and crime. What is the consequence? There are more persons living a single life: Are there more leading a virtuous life? It is time for mothers to know, that the extravagance they encourage is destructive of the virtue of their children; that all the foolish expenditures making to rush their daughters to matrimony, are, instead of answering that end, tending to destroy the institution of marriage altogether."

